# Comparison of survival between patients receiving general outpatient palliative care and patients receiving other palliative care - analysis of data of a statutory health insurance data

**DOI:** 10.1186/s12904-022-00980-x

**Published:** 2022-05-26

**Authors:** Kilson Moon, Laura Rehner, Wolfgang Hoffmann, Neeltje van den Berg

**Affiliations:** 1 Department of Epidemiology and Community Health, Institute for Community Medicine, University Medicine Greifswald, Ellernholzstr. 1-2, 17489 Greifswald, Germany; 2 Institute for Nursing Science and Interprofessional Learning, University Medicine Greifswald, Greifswald, Germany

**Keywords:** Palliative care, Survival, General outpatient palliative care (GOPC), Specialized outpatient palliative care (SOPC), Rural, Claims data

## Abstract

**Background:**

The care of palliative patients takes place as non-specialized and specialized care, in outpatient and inpatient settings. However, palliative care is largely provided as General Outpatient Palliative Care (GOPC). This study aimed to investigate whether the survival curves of GOPC patients differed from those of the more intensive palliative care modalities and whether GOPC palliative care was appropriate in terms of timing.

**Methods:**

The study is based on claims data from a large statutory health insurance. The analysis included 4177 patients who received palliative care starting in 2015 and who were fully insured 1 year before and 1 year after palliative care or until death. The probability of survival was observed for 12 months. Patients were classified into group A, which consisted of patients who received palliative care only with GOPC, and group B including patients who received inpatient or specialized outpatient palliative care. Group A was further divided into two subgroups. Patients who received GOPC on only 1 day were assigned to subgroup A1, and patients who received GOPC on two or more days were assigned to subgroup A2. The survival analysis was carried out using Kaplan-Meier curves. The median survival times were compared with the log-rank test.

**Results:**

The survival curves differed between groups A and B, except in the first quartile of the survival distribution. The median survival was significantly longer in group A (137 days, *n* = 2763) than in group B (47 days, *n* = 1424, *p* < 0.0001) and shorter in group A1 (35 days, *n* = 986) than in group A2 (217 days, *n* = 1767, *p* < 0.0001). The survival rate during the 12-month follow-up was higher in group A (42%) than in group B (11%) and lower in group A1 (38%) than in group A2 (44%).

**Conclusions:**

The results of the analysis revealed that patients who received the first palliative care shortly before death suspected insufficient care, especially patients who received GOPC for only 1 day and no further palliative care until death or 12-month follow-up. Palliative care should start as early as necessary and be continuous until the end of life.

**Supplementary Information:**

The online version contains supplementary material available at 10.1186/s12904-022-00980-x.

## Background

Palliative care is a proven approach for patients and their families facing the problems associated with life-threatening illness to improve quality of life [1]. In the German health care system, palliative care is provided at different levels of care and by different healthcare providers. Ambulatory palliative care can be delivered as General Outpatient Palliative Care (GOPC), which is provided by general practitioners (GPs) and ambulatory nursing services. Specialized Outpatient Palliative Care (SOPC) is delivered by multi-professional specialised palliative care teams (palliative medicine physicians and palliative care nurses). Inpatient palliative care is provided in designated hospices, palliative care units in hospitals, or in hospitals without a designated palliative care unit and in nursing homes [2–4].

Mecklenburg-Western Pomerania, Germany’s federal state with the lowest population density, is located in the northeastern part of the country at the Baltic Sea. Mecklenburg-Western Pomerania has a high proportion of elderly and a low number of specialized health care providers [5–7]. In rural areas, GOPC by GPs plays a major role. GOPC is an important part of home care for palliative care patients and for early recognition of palliative care needs. In addition to communication and the determination of therapeutic goals, GOPC comprises symptom control and the coordination of treatment, if necessary with the involvement of SOPC teams or inpatient palliative care [8–10].

Survival curves show the time period between the start of palliative treatment and death or the date of the last follow-up [11]. A short period of time indicates that palliative care was started shortly before death. In our recent analysis of claims data from a statutory health insurance, we observed that about two-thirds of palliative care patients in Mecklenburg-West Pomerania received only GOPC [12]. This study aimed to explore whether the survival curves of GOPC patients differed from those of the more intense palliative care modalities and to evaluate whether GOPC palliative care was appropriate in terms of timing.

## Methods

### Claims data

The analyses were based on claims data of the AOK Nordost (2015/16), which is a large statutory health insurance provider in the federal states of Berlin, Brandenburg and Mecklenburg-West Pomerania, Germany. The AOK Nordost covers more than a quarter of the total population in the federal state of Mecklenburg-Western Pomerania. The claims dataset included demographic information (age, gender, date of death) as well as inpatient and outpatient diagnoses and treatments.

### Definition of palliative treatment

Patients who received at least one palliative care service of the GOPC, SOPC, or palliative care in a hospital or hospice were included in the analysis. To determine general outpatient palliative care treatment, the codes for palliative care services from the reimbursement catalog of the statutory health insurances for outpatient care (EBM) “initial assessment of the patient’s health situation including treatment plan” (EBM codes 03370 and 04370), “additional outpatient palliative care” (EBM codes 03371, 03372, 03373, 04371, 04372 and 04373) were used. Contracts for SOPC-Teams containing the kind of care and delivery dates of the healthcare services were used to determine patients receiving SOPC services. Operations and procedures (OPS) codes for complex palliative care treatment by palliative care specialists and multidisciplinary teams on any hospital ward including intensive care units (OPS code 8-98e) and complex palliative treatment in specialized palliative care units (OPS codes 8-982) were used to determine patients with an inpatient palliative treatment in a hospital.

### Study population

The study population included 4177 patients in Mecklenburg-Western Pomerania who started their palliative care in 2015 and were insured 12 months before and 12 months after palliative care. The patients were followed up for a maximum of 12 months after the start of any palliative care. A detailed description of the sample construction was recently published [12]. Details about the sample collection are provided in Appendix A. The survival times of the included patients were observed. Patients who received GOPC only were categorized into group A. Group B included patients who received also inpatient palliative care or SOPC. Group A was further divided into two subgroups. Patients who received GOPC on only 1 day were assigned to subgroup A1, patients receiving GOPC during two or more days were assigned to subgroup A2.

### Diagnosis

The patient’s diagnoses were identified based on the International Statistical Classification of Diseases (ICD) codes (10th revision, German modification). All hospital diagnoses (primary and secondary diagnosis codes) and ambulatory diagnoses verified in at least two quarters of a single year (M2Q criterion) were used. Oncological patients were defined by ICD-10 code C00-D48.

### Statistical analyses

Descriptive statistics are presented as absolute numbers and percentages for categorical variables, and medians and interquartile ranges (IQR) for continuous data. Kaplan-Meier analysis was applied to compare survival times among groups A and B as well as groups A1 and A2. The differences between the curves were analysed with the log-rank test. All statistical analyses were conducted using SAS Software release 9.4 (Version 9.4; SAS Institute, Cary, NC, USA) and a *p*-value of < 0.05 was considered statistically significant.

### Ethics

The present study is based on a retrospective analysis of anonymised health insurance claims data available for research proposes, and therefore no formal ethics committee approval was needed [13].

## Results

A total of 4177 palliative care patients were included in the study. The median age of the patients was 81.0 years (IQR: 74.0 – 87.0) and 54.6% (*n* = 2280) were female (Table 1). During the 12-months follow-up period, 68.6% (*n* = 2866) of patients died, their median survival time was 76 days. Most of the patients (97.3%) had at least one inpatient or ambulatory diagnosis. Of these 2288 patients (56.3%) had a diagnosis of a malignant neoplasm (ICD-10 code: C00-C97). Overall, 85.7% (*n* = 3579) of the palliative care patients received at least one service including GOPC, 21.6% (*n* = 904) of the patients received at least one SOPC service. Altogether, 18.9% (*n* = 791) of the patients received inpatient palliative care in a hospital at least one time throughout the observation. In total, 2.7% (*n* = 112) of the patients were cared for in a hospice.

**Table 1:** Characteristics of the palliative care patients in 2015/16, 12-months follow-up

Number of palliative care patients, n	4177
Age (years), median (IQR)	81.0 (74.0 – 87.0)
Female, n (%)	2280 (54.6%)
Dead, n (%)	2866 (68.6%)
- median survival days (95% CI)	76.0 (70.0, 83.0)
Inpatient or ambulatory diagnoses, n (%)	4062 (97,3%)
- oncological patients, n (%)	2477 (61.0%)
Number of patients (n, %) with services in	
- GOPC	3579 (85,7%)
- SOPC	904 (21,6%)
- Hospital	791 (18,9%)
- Hospice	112 (2,7%)
number of care days, median (IQR)	
- GOPC	2.0 (1.0 – 4.0)
- SOPC	17.0 (7.0 – 49.0)
- Hospital	14.0 (9.0 – 23.0)
- Hospice	37.5 (20.5 – 70.0)

*IQR* interquartile range (25th quartile – 75th quartile), *CI* confidence interval, *GOPC* General Outpatient Palliative Care, *SOPC* Specialized Outpatient Palliative Care

**Fig. 1:**
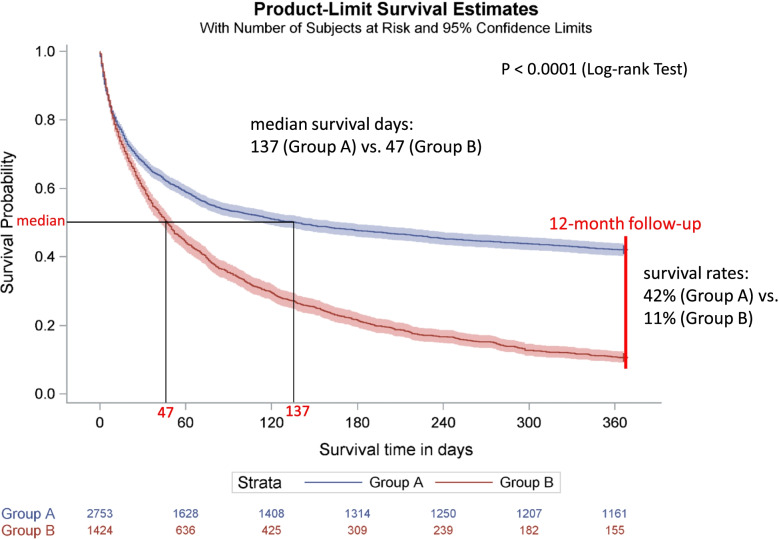
Kaplan-Meier Curve of palliative care patients (12-month follow-up): Group A vs. Group B. Group A: patients who received only General Outpatient Palliative Care. Group B: patients who received also inpatient or Specialized Outpatient Palliative Care

About two-thirds of the palliative care patients (*n* = 2753) received only GOPC during the 12-month follow-up period and no further inpatient palliative care or SOPC (group A). One-third of the patients (*n* = 1424) received in addition to GOPC inpatient palliative care and/ or SOPC (group B) (Table 2). The median age in group A was 82.0 (IQR: 75.0 – 88.0) years and in group B 79.0 (IQR: 70.5 – 84.0) years. During the 12-months follow-up, 57.9% (*n* = 1595) of patients from group A and 89.3% (*n* = 1271) from group B died. The rate of oncological patients was lower in group A (50.6%) than in group B (80.5%). A Kaplan–Meier curve comparing the survival times of groups A and B is shown in Fig.1. The survival time curves differed between group A and group B, except in the first quartile of the survival distribution (survival probability from 1 to 0.75). The median survival time was significantly longer in group A (137 days) than in group B (47 days, log-rank test: *p* < 0.0001).

**Table 2:** Characteristics and survival times of the palliative care patients in group A and B

	Group A	Group B
Number of palliative care patients	2753	1424
Age, years, median (IQR)	82.0 (75.0 – 88.0)	79.0 (70.5 – 84.0)
Female, n (%)	1549 (56.3%)	731 (51.3%)
Dead, n (%)	1595 (57.9%)	1271 (89.3%)
- median number of survival days (95% CI)	137.0 (113.0, 172.0)	47.0 (42.0, 52.0)
Inpatient or outpatient diagnoses, n (%)	2651 (96.3%)	1411 (99.1%)
- oncological patients, n (%)	1341 (50.6%)	1136 (80.5%)

*IQR* interquartile range (25th quartile – 75th quartile), *CI* confidence interval

Group A: patients receiving only General Outpatient Palliative Care

Group B: patients receiving inpatient or Specialized Outpatient Palliative Care

The Kaplan-Meier curves separate for oncological patients and patients with other diagnoses (non-oncological patients) are shown in Appendix B. In oncological and non-oncological patients, survival time was significantly shorter in group A compared to group B (respectively, log-rank test: *p* < 0.0001), and the difference in survival times was greater in oncological patients than in non-oncological patients.

**Fig. 2:**
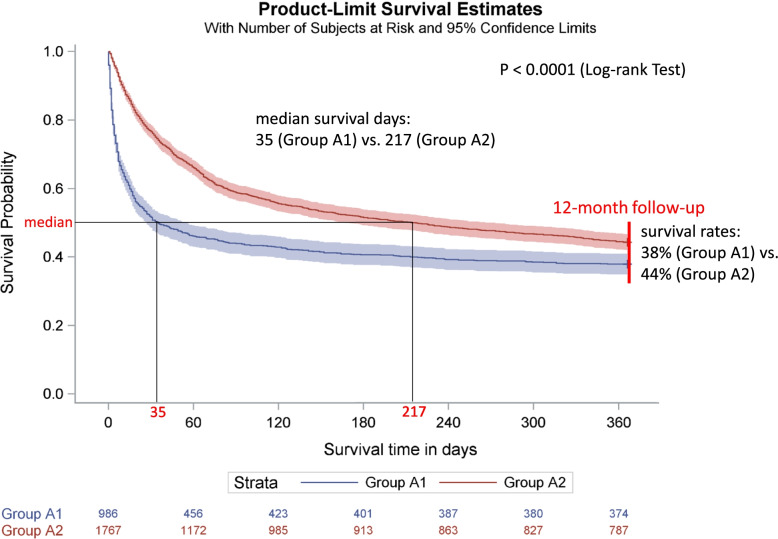
Kaplan-Meier Curve of palliative care patients (12-month follow-up): Group A1 vs. Group A2 Group A1: patients receiving only General Outpatient Palliative Care, treatment = 1 day. Group A2: patients receiving only General Outpatient Palliative Care, treatment > 1 day

Patients in group A1, who accounted for 36% (*n* = 986) of group A (*n* = 2753), received only 1 day of GOPC, and patients in group A2, who accounted for 64% (*n* = 1767) of group A, received more than 1 day of GOPC (Table. 3). The median age of both groups is 82.0 years. During the 12-month follow-up, 62.1% (*n* = 612) of patients from group A1 and 55.6% (*n* = 983) from group A2 died. The survival rate was lower in group A1 (37.9%) than in group A2 (44.4%). The rate of oncological patients was lower in group A1 (46.8%) than in group A2 (52.7%). A Kaplan–Meier curve comparing the survival times of the groups A1 and A2 is shown in Fig.2. The survival curves differed between the two groups. The median survival time was significantly shorter in group A1 (35 days) compared to group A2 (217 days, log-rank test: *p* < 0.0001). The Kaplan-Meier curves separated for oncological patients and non-oncological patients are presented in Appendix C. Survival time was also significantly shorter in group A1 compared to group A2 both in oncological patients (log-rank test: *p* = 0.0013) and in non-oncological patients (log-rank test: *p* < 0.0001), however, the difference in survival times was smaller in oncological patients than in non-oncological patients.

**Table 3:** Characteristics and survival time of the palliative care patients in group A1 and A2

	Group A1	Group A2
Number of palliative care patients	986	1767
Age (years), median (IQR)	82.0 (75.0 – 88.0)	82.0 (75.0 – 88.0)
Female, n (%)	573 (58.1%)	976 (55.2%)
Dead, n (%)	612 (62.1%)	983 (55.6%)
- median number of survival days (95% CI)	35.0 (27.0, 56.0)	217.0 (167.0, 273.0)
Inpatient or outpatient diagnoses, n (%)	936 (94.9%)	1715 (97.1%)
- oncological patients, n (%)	438 (46.8%)	903 (52.7%)
Number of GOPC days, median (IQR)	1.0 (1.0 – 1.0)	3.0 (2.0 – 5.0)

*IQR* interquartile range (25th quartile – 75th quartile), *CI* confidence interval, *GOPC* General Outpatient Palliative Care

Group A1: patients receiving only General Outpatient Palliative Care, treatment = 1 day

Group A2: patients receiving only General Outpatient Palliative Care, treatment > 1 day

## Discussion

In general, palliative care patients who received only GOPC (group A) had a longer survival time than patients who received also inpatient palliative care or SOPC (group B). This may indicate that patients in group A had less severe symptoms or a better medical condition. In contrast, patients in group B may have had a worse medical condition that required a higher level of care with specialized palliative care. The patients in group B more often had a diagnosis of cancer and the survival rate after 12 months of follow-up was lower than the patients in group A. In the Kaplan-Meier survival curves, the difference between the two groups was statistically significant. The curves also reveal how many patients died shortly after starting palliative care. The survival time of patients who died soon after the beginning of palliative care did not differ between the two groups. About a quarter of the patients in both groups died within 2 weeks after starting palliative care. In other words, the patients received palliative care only shortly before their death. This could mean that palliative care started too late for some of these patients.

Palliative care aims to improve the quality of life of patients and their families by preventing and alleviating suffering through early identification and impeccable assessment and treatment of pain and other physical, psychosocial and spiritual problems [1]. Palliative care offers patients support so that they can live as actively as possible with a decent quality of life until their death. Therefore, palliative care should start as early as necessary rather than just shortly before death. Studies suggest that timely palliative care can improve the quality of life of patients with advanced-stage disease [14–18]. Early referral to palliative care for patients can facilitate appropriate monitoring and treatment of symptoms, longitudinal psychosocial support, counselling, and a gradual transition of care [14–16]. Besides, early palliative care can provide further benefits to the health care system by ameliorating the caregiver distress and health care costs associated with aggressive end-of-life care [17, 18].

Primary care providers, such as GPs, provide most palliative care in Germany. GPs play a key role in determining the need for palliative care and requesting a palliative care consultation, as well as coordinating referrals to palliative care specialists. Although many GPs consider palliative care an essential part of their work, knowledge of palliative care and the structures of specialized palliative care were limited among GPs due to a lack of qualifications and experience in palliative care [19–21]. The present data, however, do not provide any information on the quality of palliative care provided by GPs.

The present study showed that about two-thirds of palliative care patients received only GOPC provided by GPs and no inpatient palliative care or SOPC services (group A). One-third of them received only 1 day of GOPC. The survival time of these patients (group A1) was shorter than that of patients receiving more than 1 day of GOPC (group A2). In contrast to group A2, in which half of the patients died within 7 months of starting GOPC, half of the patients in group A1 died already within 1 month. Moreover, one-third of the patients in group A1 died within 1 week after their first palliative care measure. This indicates a rather late start of palliative care for this subgroup of patients who received only GOPC. However, during the study period, a large proportion of patients in group A1 survived after starting palliative care and received no further palliative care. This may be due either to the fact that no further palliative care was required for the patients or that the patients’ initial assessment as palliative care patients by GPs was inadequate. It is a major challenge for GPs to ensure the assessment of patients who need GOPC or are to be referred to SOPC.

In one federal state in Germany (North Rhine-Westphalia), this problem is addressed by an innovative design of palliative care, whereby the basic concept is consciously designed to integrate GOPC and SOPC structures in one contract. Palliative care is based on cooperation between palliative physicians and GPs. Palliative physicians and coordinators are organized in this region at a regional level in palliative medicine consultation services. The coordinators of these services organize the cooperation between GPs, clinics, nursing homes as well as other facilities and the palliative physicians [22, 23]. Furthermore, since 2017, a new reimbursement code for physicians “specially qualified and coordinated palliative care” was introduced in Germany. The new reimbursement code is intended to facilitate transitions between curative treatment, GOPC and SOPC [24].

This study shows that palliative care in the federal state Mecklenburg-Western Pomerania in German may not be appropriate in a part of the cases. The results also indicate that general palliative care may not be continuous for many palliative care patients. Palliative care in the whole of Germany is organized similarly as in Mecklenburg-Western Pomerania. Although the utilization of palliative care in Germany varies from region to region [25], the results are probably transferable to other rural regions in Germany. Further research should be applied on the early initiation of palliative care and continuity of palliative care.

It should be taken into account that the claims data do not provide any information on palliative care provided by ambulatory nursing services, which probably also make an important contribution to the provision of GOPC. However, little is known about the nature and extent of palliative care delivered by nursing services. Further research on this issue is needed.

### Strengths and limitations

A strength of the study lies in the health insurance data, which includes inpatient and outpatient data. This data allows studying the course of patients through different sectors of the health care system. However, a limitation of claims data is that they were collected for reimbursement purposes and may not fully and accurately reflect the individual health situation of the patients. Further limitations are that the patient’s home situation is not reflected and the need for palliative care, especially GOPC, cannot be determined. Although a large part of the population in the study region is insured by AOK-Nordost, the results of the analysis may not fully extend to the entire population of palliative care patients. Another limitation is that Group B includes patients who received palliative care in the hospital and for whom operations and procedures codes have been defined, as there are no palliative-specific Diagnosis-Related Groups. However, hospital palliative care may be reimbursed based on the Diagnosis-Related Groups system, i.e., without operations and procedures codes. In this case, these patients are overlooked.

## Conclusion

The longer survival time of patients with GOPC may be an indication that this group had less advanced disease with less severe symptoms and that the kind of palliative care was probably appropriate. However, subgroups of patients received their first palliative treatment shortly before they died or did not receive any further palliative care after starting palliative care. Palliative care should start as early as necessary and continue until the end of life to improve the quality of life of patients.

## Supplementary Information

**Additional file 1: Appendix A**. Flow-chart of patient selection.

**Additional file 2: Appendix B**. Kaplan-Meier Curve of palliative care oncology and non-oncology patients (12-month follow-up): Group A vs. Group B. Group A: patients who received only General Outpatient Palliative Care. Group B: patients who received also inpatient or Specialized Outpatient Palliative Care.

**Additional file 3: Appendix C**. Kaplan-Meier Curve of palliative care oncology and non-oncology patients (12-month follow-up): Group A1 vs. Group A2 Group A1: patients receiving only General Outpatient Palliative Care, treatment = 1 day. Group A2: patients receiving only General Outpatient Palliative Care, treatment > 1 day.

## Data Availability

The project is funded by the Ministry of Economic Affairs, Labour and Health of Mecklenburg-Western Pomerania, Germany.
