# Supplementary figures and images for: Comparison of survival between patients receiving general outpatient palliative care and patients receiving other palliative care - analysis of data of a statutory health insurance data
Source: BMC Palliat Care. 2022 May 26;21:88. doi: 10.1186/s12904-022-00980-x (PMC9134662; doi:10.1186/s12904-022-00980-x)

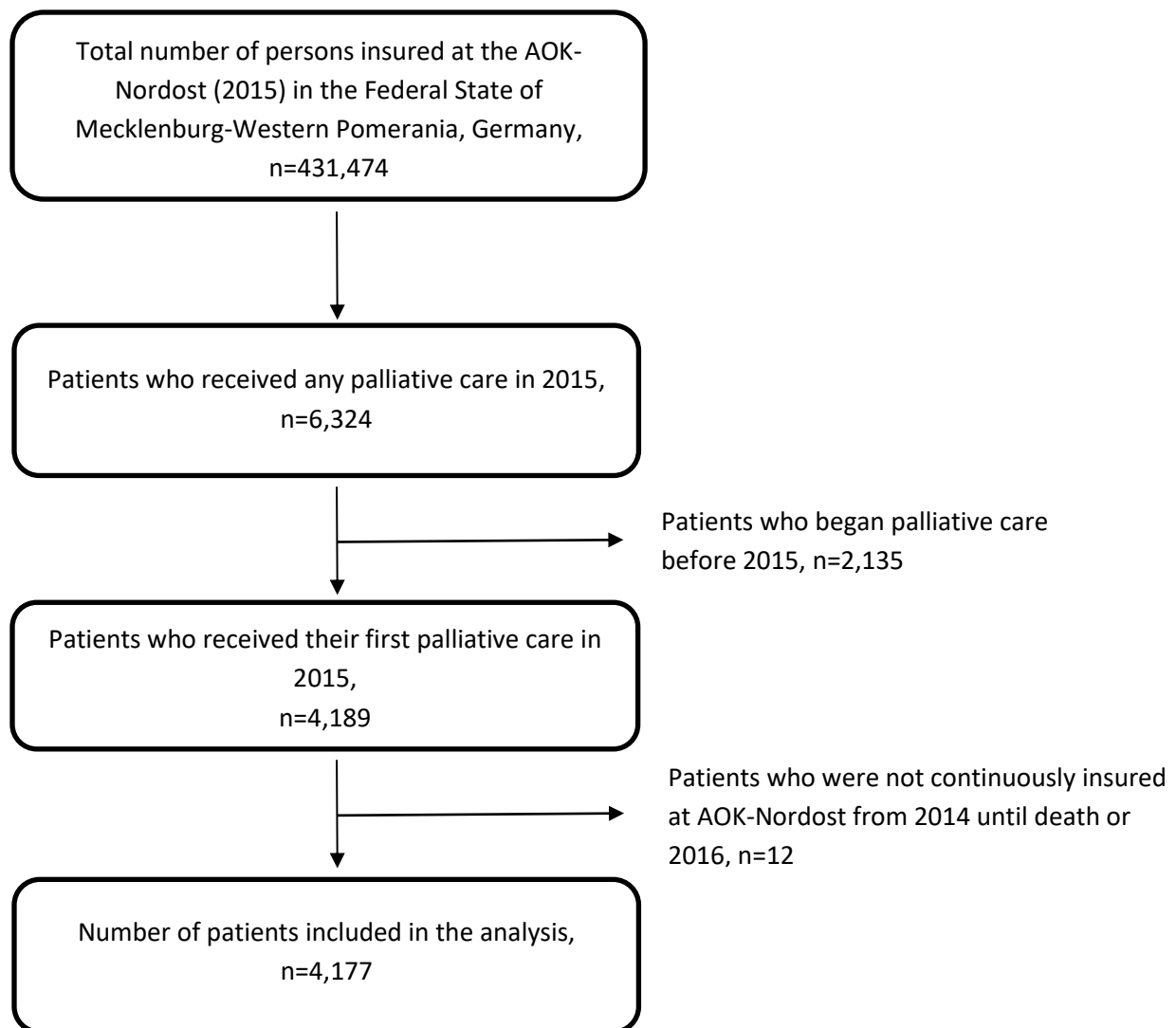

Supplement: Supplementary file 1 — Additional file 1: Appendix A. Flow-chart of patient selection. [file 12904_2022_980_MOESM1_ESM.pdf]
